# Cross-sectional and longitudinal analysis of cancer vaccination trials registered on the US Clinical Trials Database demonstrates paucity of immunological trial endpoints and decline in registration since 2008

**DOI:** 10.2147/DDDT.S65963

**Published:** 2014-09-27

**Authors:** Liangjian Lu, Haixi Yan, Vijay Shyam-Sundar, Tobias Janowitz

**Affiliations:** 1 School of Clinical Medicine, University of Cambridge, Cambridge, UK; 2 Cancer Research UK, Cambridge Institute, Li Ka Shing Centre, University of Cambridge, Cambridge, UK

**Keywords:** cancer vaccination, cancer prevention, clinical trials, translational trial endpoints, immunotherapy

## Abstract

**Introduction:**

Cancer vaccination has been researched as a means of treating and preventing cancer, but successful translational efforts yielding clinical therapeutics have been limited. Numerous reasons have been offered in explanation, pertaining both to the vaccine formulation, and the clinical trial methodology used. This study aims to characterize the tumor vaccine clinical trial landscape quantitatively, and explore the possible validity of the offered explanations including the translational obstacles posed by the current common endpoints.

**Methods:**

We performed a detailed cross-sectional and longitudinal analysis of tumor vaccine trials (n=955) registered in the US Clinical Trials database.

**Results:**

The number of tumor vaccine trials initiated per annum has declined 30% since a peak in 2008. In terms of vaccine formulation, 25% of trials use tumor cell/lysate preparations; whereas, 73% of trials vaccinate subjects against defined protein/peptide antigens. Also, 68% of trials do not use vectors for antigen delivery. Both these characteristics of tumor vaccines have remained unchanged since 1996. The top five types of cancer studied are: melanoma (22.6%); cervical cancer (13.0%); breast cancer (11.3%); lung cancer (9.5%); and prostate cancer (9.4%). In addition, 86% of the trials are performed where there is established disease rather than prophylactically, of which 67% are performed exclusively in the adjuvant setting. Also, 42% of Phase II trials do not measure any survival-related endpoint, and only 23% of Phase III trials assess the immune response to vaccination.

**Conclusion:**

The clinical trial effort in tumor vaccination is declining, necessitating a greater urgency in identifying and removing the obstacles to clinical translation. These obstacles may include: 1) vaccination against a small range of antigens; 2) naked delivery of antigen; 3) investigation of less immunogenic cancer types; and 4) investigation in the setting of established disease. In addition, the prevalence of late phase failure may be due to inadequate assessment of survival-related endpoints in Phase II trials. The clinical trial development of tumor vaccines should include mechanism-based translational endpoints, as well as the discovery of immune biomarkers with which to stratify, monitor, and prognosticate patients.

## Introduction

Numerous lines of evidence suggest that tumor cells can be recognized and eliminated by the immune system.^1^ On April 29, 2010, sipuleucel-T (Dendreon Corporation, Seattle, WA, USA) received US Food and Drug Administration approval for use in minimally symptomatic castration-resistant prostate cancer.^2^,^3^ This was the first therapeutic cancer vaccine to obtain the US Food and Drug Administration approval, a landmark success in the field of cancer vaccines, and – at the same time – a reminder of the hitherto low therapeutic yield^4^ of the field. In the context of the recent successes of immunomodulatory strategies in Phase II and Phase III trials,^5^,^6^ it is questionable whether cancer vaccines represent an optimal approach for inducing greater immunological control of established disease. It can be argued that a cancer vaccine will have greater efficacy if antigen presentation and the afferent arm of the immune system are impaired, while the immunomodulatory strategies directed at the T-cell checkpoints and signaling will be more effective if the efferent arm of the immune system, especially T-cell function, is impaired.^1^,^7^ A definitive answer that instructs clinical translational efforts^8^ is some years away.

Over the years, numerous reviews have examined the obstacles in translating vaccinations that show promise in preclinical research into clinical practice. To understand to what extent these obstacles are reflected in the clinical trial effort as a whole, we characterized the trial landscape by analyzing the registered trials on the US trial database (http://www.clinicaltrials.gov). We also investigate if the current trial methodology includes the translational research endpoints that can inform vaccine development in the future.

These aforementioned obstacles can be broadly divided into two areas: 1) the nature of the vaccination approach; and 2) the existing clinical trial methodology.^9^

The therapeutic intervention in cancer vaccine trials consists of a particular formulation of tumor-associated antigens (TAAs) delivered together with adjuvant(s). There remains considerable uncertainty as to the optimal formulation of TAAs. Irradiated tumor cells or tumor cell lysates have been suggested to be superior antigen formulations, as they stimulate an immune response against diverse tumor antigens making immune escape less likely, compared to the formulations containing one or a few recombinant TAAs.^10^

It is also important to consider the growing evidence for the necessity of stimulating cytotoxic T-lymphocytes (CTLs) to produce a tumor response.^11^ CTLs express the cluster of differentiation 8 (CD8) T-cell receptor (TCR) coreceptor and are major histocompatibility complex class I-restricted.^12^ Consequently, this suggests that TAAs need to be delivered packaged – eg, in viral vectors – as opposed to naked, or alternatively pulsed into antigen presenting cells (APCs) ex vivo before infusion.

Besides the antigen formulation, there is similarly little clarity as to the optimal adjuvants.^4^,^13^ Classically, adjuvants stimulate APC maturation, inducing the expression of costimulatory molecules and proinflammatory cytokines, which are necessary for the complete activation of T-cells.^10^ Increasingly, with the characterization of immune checkpoints that prevent a spontaneously effective antitumor immune response in cancer patients, eg, the engagement of T-cell cytotoxic T-lymphocyte antigen 4 and programmed death 1 (PD-1) in the immunosuppressive tumor microenvironment, adjuvants may more broadly include biologicals that relieve these checkpoints, such as ipilimumab and nivolumab, respectively.^14^,^15^

Besides the actual vaccination approach, another broad area of criticism is the existing clinical trial methodology. Both the suitability of patient enrollment criteria used in cancer vaccine trials – as well as the methods of assessing the therapeutic effects of the vaccine – are thought to be implicated in low translational yields from cancer vaccine trials.

Some tumors – for instance, melanomas and clear cell renal cell carcinomas – are considered more immunogenic than others, as evidenced by the tumors being frequently infiltrated by the CD8+ T-lymphocytes, which correlates with favorable prognosis, and occasionally undergoing spontaneous regression.^14^,^16^–^21^ These tumor types may be more amenable to immunotherapy, with tumor vaccination at least serving to trigger the generation of an antitumor immune response in the proportion of patients who fail to do so spontaneously.^22^ Thus, the clinical trial efforts focused on other tumor types less tractable to immunotherapy could explain, in part, the failures of translational efforts.^4^,^14^

In advanced disease, multiple redundant mechanisms of immune escape are present in the tumor microenvironment, and there may be a global systemic dysfunction of T-cells as well, perhaps due to chronic nonproductive antigenic stimulation.^16^,^23^ This may explain poor vaccine efficacy in advanced cancers. Maybe cancer vaccines should be mainly trialled in the prophylactic or, at the very least adjuvant, setting.^8^,^23^,^24^ Cancers with well-established precursor lesions – eg, colonic adenomas and pancreatic intraepithelial neoplasia – may be good candidates for prophylactic vaccination, as they allow a high-risk group to be targeted for vaccination.^25^,^26^

Another area of intense discussion concerns the suitability of trial endpoints, which were originally formulated to assess the efficacy of cytotoxic chemotherapies. Immune responses have different kinetics from cytotoxic chemotherapies. They may also appear differently in the conventional trial assessment methods, for instance, the infiltration of the tumor by lymphocytes leading to apparent radiological progression.^9^,^27^ This calls into question the validity of survival measures, such as the radiologically assessed progression free survival (PFS) being used as the primary endpoint of clinical trials, in spite of its acceptance by regulatory authorities.^28^

Many of the aforementioned reasons proffered to explain the poor therapeutic yield of tumor vaccines are based on a careful consideration of preclinical data as well as the published results of certain clinical trials. It remains an open question, whether such analyses are valid across the entire spectrum of cancer vaccine interventional trials, so as to have sufficient explanatory power to account for the limited translational success of the field in general. It is also still uncertain whether these analyses have encouraged, or at least correspond with, changes across the field. Consequently, these analyses will be greatly complemented by a cross-sectional and longitudinal study of the tumor vaccine clinical trial landscape, which we have undertaken and report here.

## Materials and methods

### Database creation and analysis

This study is a cross-sectional and longitudinal study of interventional cancer vaccine trials registered on the Clinical Trials Database (http://www.clinicaltrials.gov). It has been conducted and reported according to the Strengthening the Reporting of Observational Studies in Epidemiology (STROBE) criteria.^29^ On June 19, 2013, using the advanced search function, trials with the terms “cancer” in their list of conditions, and “vaccine” in their list of interventions, as well as registered as being of an “interventional” study type, were selected. Interventional studies refer to those in which an intervention of any type, including drugs, procedures, and rehabilitation strategies, was investigated, as opposed to purely observational studies.^30^ Study details were downloaded as datasets for review using Microsoft Excel (Microsoft Corporation, Redmond, WA, USA).

**Figure 1:**
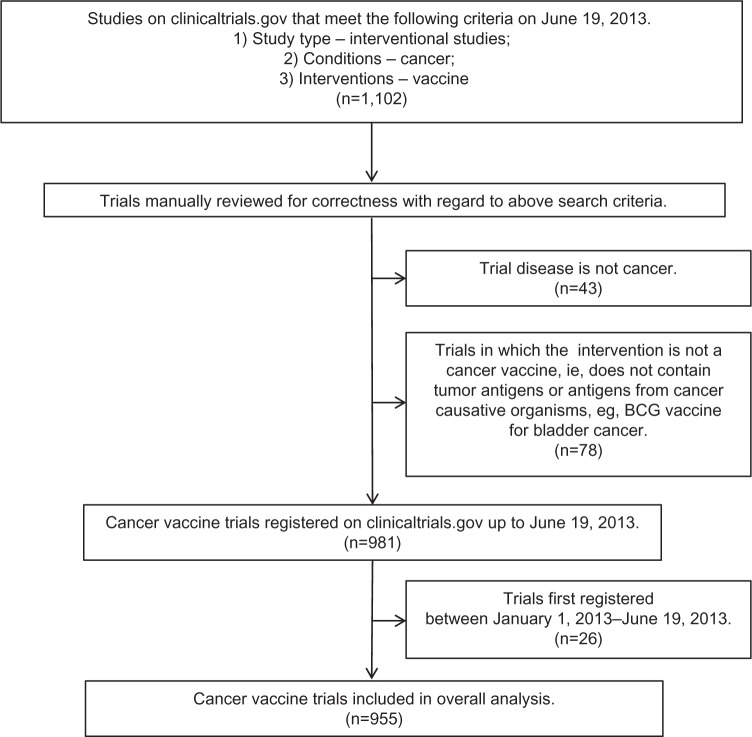
Flow diagram of database creation and manual review of trials. **Note:** 955 trials in total are included in the overall analysis. **Abbreviation:** BCG, Bacillus Calmette–Guérin.

All studies were manually checked to ensure that they were trials of cancer vaccines in the prevention or therapy of cancer. This led to the exclusion of 43 trials in which the primary condition being investigated was not cancer, eg, influenza vaccinations in children with cancer (NCT00022035), as well as the exclusion of a further 78 trials in which the intervention was not a cancer vaccine per se, eg, the Bacillus Calmette–Guérin vaccine for bladder cancer (NCT00427570). All suitable trials registered before 2013 were included, yielding a total of 955 trials for analysis (Figure 1).

The data that were downloaded into Microsoft Excel were not edited. As a result, a proportion of the trials was missing certain data fields. Incomplete registration details are a known limitation of trial database entries.^31^ The respective trials were excluded from the relevant parts of the analysis,^31^ leading to a total trial count of <955 for most subsections. Total trial counts are always specified for each analysis performed. For certain analyses, such as the clinical setting of vaccination, the downloaded datasets provided insufficient information. In these cases, the online registration entry of the trial on the Clinical Trials Database was referred to for further details. For the analysis of enrollment numbers, comparison with interventional cancer trials in general, ie, all interventions, was needed. The relevant trial data were downloaded from the Clinical Trials Database on June 19, 2013 with the following criteria: 1) study type – interventional studies; and 2) conditions – cancer. This yielded a total of 30,859 trials. These data were not subject to manual review.

## Statistical analysis

All averages are given as mean ± standard error of mean, unless otherwise stated. For selected longitudinal series, linear regression analysis was performed. For comparison of means and medians, the Student’s *t*-test and the Wilcoxon rank-sum test were performed, respectively. Also, *P*<0.05 was taken to indicate statistical significance.

## Results

### Overall trial characteristics

**Figure 2:**
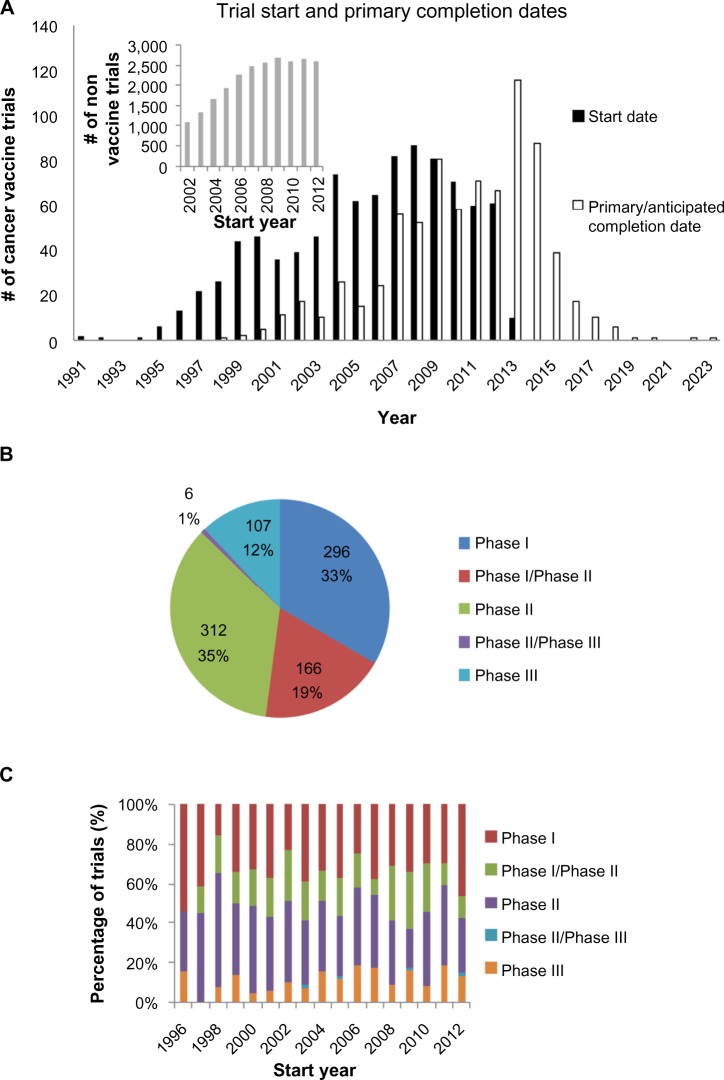
Overall trial characteristics. **Notes:** (**A**) Start and primary completion dates. The # of trials initiated is on the decline, with a clear peak in 2008. This decline is not observed for nonvaccine cancer interventional trials (inset). (**B**) Cross-sectional and (**C**) longitudinal analysis of trial phase, showing only data from Phase I to Phase III trials. The distribution of trials between phases is relatively constant over time. Analysis restricted to years with at least ten registered trials.

Of the 955 trials included in the overall analysis, data for the trial start date and the trial primary completion date were available for 935 trials and 776 trials, respectively. A longitudinal analysis of trial start dates (Figure 2A) reveals a decline in the number of clinical trials initiated, with the count declining 30% from a peak of 87 in 2008 to 61 in 2012. On a per annum basis, the number of vaccine trials decreased on average by 6.5 each year from 2008 onward (*P*<0.01). The corresponding peak in trial completion is in 2013 (Figure 2A). This is broadly in line with a calculated mean trial duration of 4.0±0.1 years (n=776). In contrast, the counts of trial start dates for the nonvaccine interventional cancer vaccine trials have stayed approximately constant from 2008–2012 (*P*=0.68). Relative to 2008, the trial counts for subsequent years are significantly different (*P*=0.02), when comparing between vaccine and nonvaccine trials.

Phase data were available for 887 Phase I–Phase III trials. There are similar numbers of Phase I and Phase II trials, which together account for 88% of trials conducted (Figure 2B). This relative proportion of clinical trials has stayed fairly constant since 1996 (Figure 2C).

## Nature of vaccination approach

**Figure 3:**
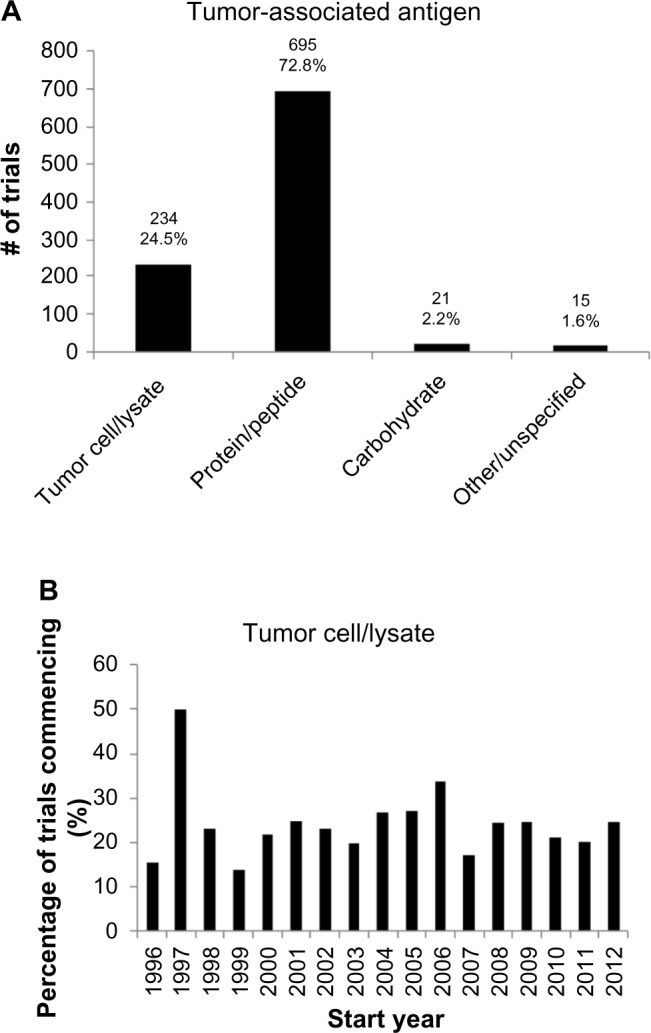
Types of antigen in vaccine formulation. **Notes:** (**A**) Cross-sectional analysis of tumor-associated antigens in clinical trials. Approximately three times as many trials vaccinate subjects against a given number of defined protein antigens, rather than a larger number of undefined tumor antigens derived from tumor cells or tumor lysates. (**B**) Proportion of trials using tumor cell/lysate formulations over time. The proportion is relatively constant with time.

A cross-sectional analysis of the antigen formulation used in the 955 trials was performed as shown in Figure 3. In 75% of the trials, the tumor antigens vaccinated against were a relatively small number of specified protein or carbohydrate antigens; whereas, in 25% of the trials, patients were exposed to a wide range of tumor-associated antigens via administration of tumor cells or their lysates (Figure 3A). This proportion has remained relatively stable across time, with an annual mean of 24.2%±2.0% from 1996–2012 (Figure 3B).

**Figure 4:**
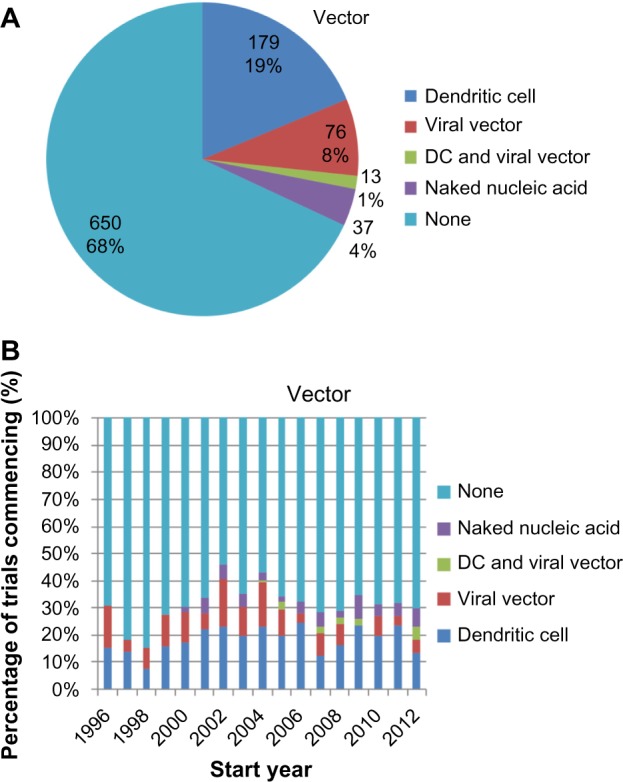
Use of vectors in vaccine formulation. **Notes:** (**A**) Cross-sectional and (**B**) longitudinal analysis of the vector used in the delivery of tumor-associated antigens. The majority of trials use vaccine formulations that do not incorporate vectors, and there is no trend toward increased vector utilization. **Abbreviation:** DC, dendritic cell vaccine.

The majority (68%) of the trials involved the administration of naked antigen (Figure 4A). This proportion has stayed relatively constant across the time, with an annual mean of 68.9%±1.8% from 1996–2012 (Figure 4B). The remaining 32% of the trials adopted vectors that comprised: dendritic cells (20%); viral vectors (9%), especially poxviruses and adenoviruses; and naked nucleic acids (4%), including unpackaged deoxyribonucleic acid, ribonucleic acid, and plasmids. Notably, even though only 1% of all the trials employed anti-idiotype vaccines, 33% of the trials in which carbohydrate antigens were vaccinated against used anti-idiotype vaccines. This reveals the utility of the anti-idiotype vaccines in specifying carbohydrate antigens, which are generally less immunogenic than protein antigens.

By an inspection of the data, we observed that almost all trials included the administration of adjuvants in the intervention under study. However, a wide range of adjuvants was used, rendering systematic categorization impossible and inappropriate. To demonstrate this spectrum of adjuvants, we chose to examine those employed in Phase III trials initiated within the last 5 years (n=40; Table S1), as these experimental vaccines are likely to have the greatest near-term clinical significance. As shown in Table 1, a total of 15 vaccines were studied in 40 trials. These vaccines utilized eight different adjuvants. There was, thus, no apparent preference for any adjuvant. Even among the four human papillomavirus (HPV) vaccines analyzed, all of which comprised viruslike particles that were made up of L1 capsid protein of various HPV strains, different adjuvants were used. For example, one used alum (aluminum hydroxide); another two, amorphous aluminum hydroxyphosphate sulfate; and a final one, a combination of alum and monophosphoryl lipid A.

## Existing trial methodology: patient characteristics

In addition, 1,178 instances of various cancers were studied across 955 trials; 28 trials did not specify, or incompletely specified, the type of cancer studied. Of the remaining 927 trials, 860 (93%) studied one cancer type. Many of the cancers that did not adequately specify cancer type had – instead – inclusion criteria based on the tumor antigen expression, eg, overexpression of vaccine antigen.

**Figure 5:**
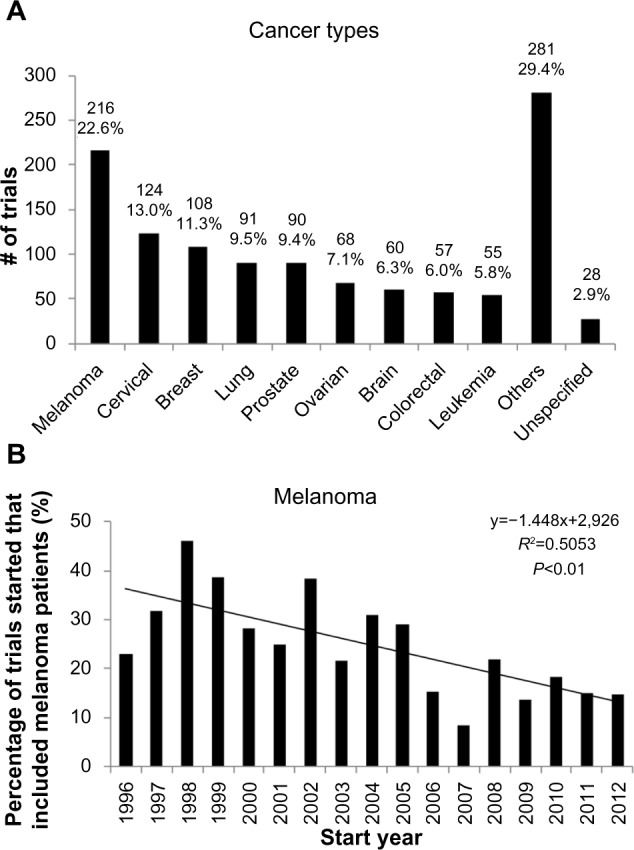
Cancer types in cancer vaccine trials. **Notes:** (**A**) Distribution of cancer types under study across trials. The top five cancers comprise common cancers like breast, lung, and prostate, as well as cancers that are thought to be particularly relevant to immune/vaccination therapy. (**B**) Longitudinal analysis of trials which study vaccination in melanoma. This reveals a clear decline over time.

The distribution of cancer types across trials is shown in Figure 5A, with the nine cancer types studied in 5% or more of trials being reflected. The top five cancer types were: melanoma (22.6%); cervical cancer (13.0%); breast cancer (11.3%); lung cancer (9.5%); and prostate cancer (9.4%). The other cancer type thought to be immunogenic, renal cancer, was studied in only 36 (3.8%) trials. While the predominance of melanoma is striking, there is a statistically significant decrease in the percentage of trials in which it is studied (Figure 5B).

**Figure 6:**
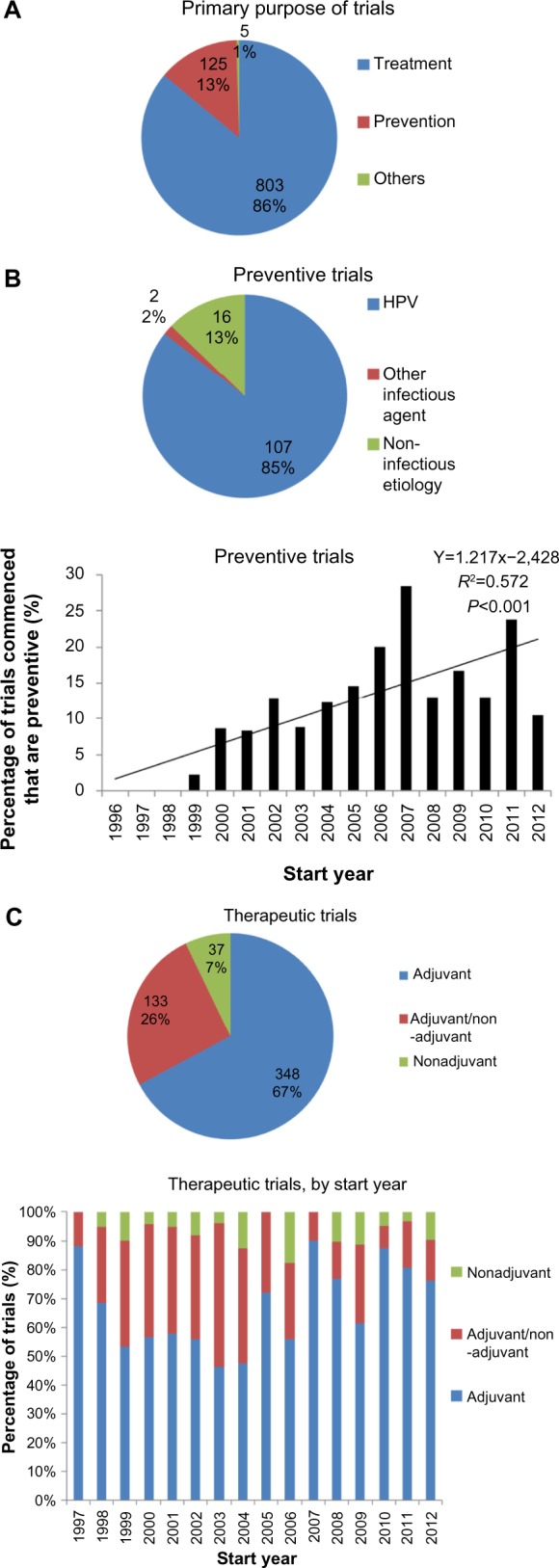
Clinical setting of cancer vaccine trials. **Notes:** (**A**) Primary purpose of all cancer vaccine trials, showing that a minority of trials involve preventive vaccines. (**B**) Preventive cancer trials. These are mainly for cancers with known infectious etiologies (upper) and the proportion of preventive cancer trials as a proportion of all trials may be increasing (lower). (**C**) Therapeutic cancer trials. Cross-sectional analysis (upper) and longitudinal analysis (lower) of cancer vaccine trials in the therapeutic setting reveals that the majority have an adjuvant focus. **Abbreviation:** HPV, human papillomavirus.

Data on the primary purpose were available for 933 of the 955 trials included. Overall, the majority of trials investigated therapeutic cancer vaccines, although a minority of the trials (13%) was performed in the preventive setting. Most of the preventive trials involved cancer types with a suggested or proven infectious etiology, especially HPV in cervical cancer (Figure 6B). Since 1996, there is a trend toward a higher proportion of preventive cancer vaccine trials (Figure 6B).

## Existing trial methodology: endpoints

**Figure 7:**
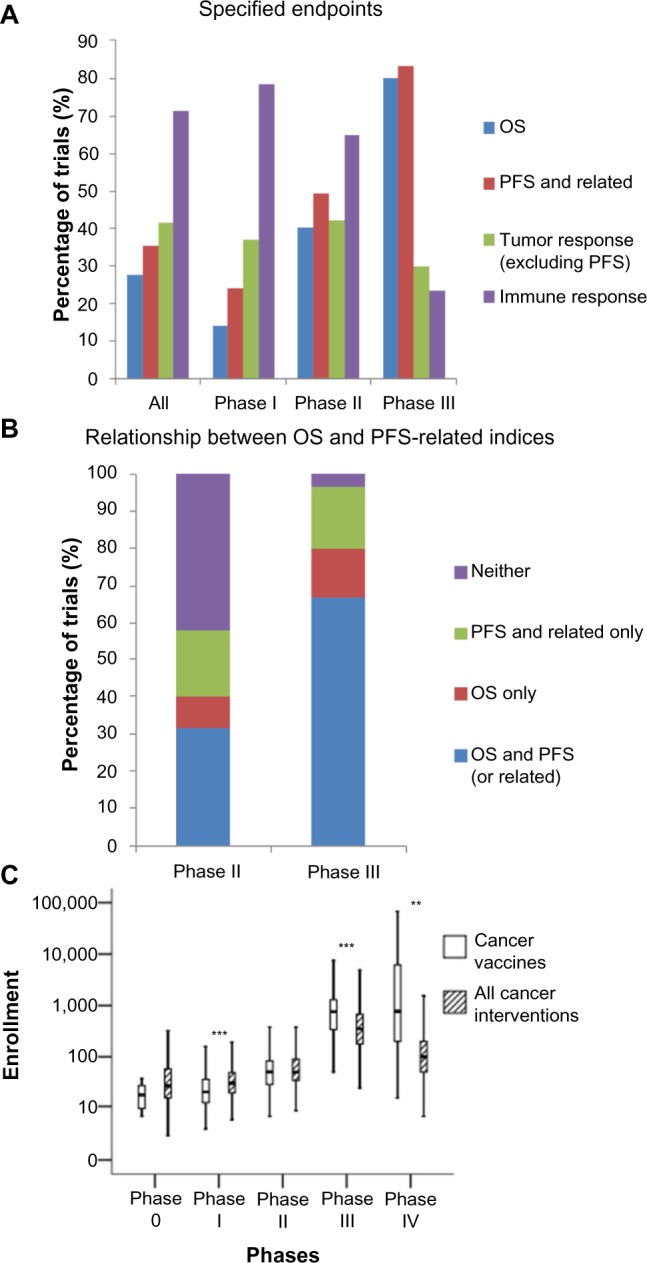
Trial methodology. **Notes:** (**A**) Relative proportion of trial endpoints by trial phase. Immune response is only measured in a small minority of Phase III trials. (**B**) A cross-sectional analysis of the adoption of survival-related endpoints. A sizable proportion of Phase II trials do not incorporate any survival-related endpoint, and some Phase III trials do not assess OS. (**C**) Absolute enrollment numbers of cancer vaccine trials compared to all cancer trials. ** and *** refer to *P*<0.01 and *P*<0.001, respectively. Compared to all interventional cancer trials, Phase I cancer vaccine trials are smaller, and Phase III trials are larger. **Abbreviations:** PFS, progression free survival; OS, overall survival.

Considering only therapeutic (as oppose to preventive) trials, endpoint data were available for 637 of the 803 therapeutic trials. There is a decrease in the proportion of the trials assessing the immune response, eg, through antibody titers and T-cell enzyme-linked immunosorbent spot assays, declining from 78% of Phase I trials to 23% of Phase III trials (Figure 7A).

Examining the use of survival-related endpoints, such as overall survival (OS) and PFS, more closely (Figure 7B), 42% of the Phase II trials did not include any of these endpoints in the assessment of therapeutic efficacy. In the Phase III trials, 17% of trials did not measure OS. Instead, they relied on other survival-related endpoints, such as PFS (Figure 7B).

Trial size was also assessed in terms of enrollment numbers. Enrollment numbers were available for 838 of the 955 trials, but suspended (nine), withdrawn (61), and terminated (20) trials were further excluded due to incomplete enrollment, thus yielding 748 trials in this analysis. Data were analyzed by phase, with data from mixed-phase trials discarded. As expected, trial enrollment numbers increase with the trial phase (Figure 7C). Comparing trial enrollment numbers of the cancer vaccine trials with those of cancer trials in general (n=25,638), trial size is significantly smaller in Phase I cancer vaccine trials, but significantly larger in Phase III and Phase IV cancer vaccine trials, compared to all cancer trials. The former may be due to the reduced toxicity of cancer vaccines compared to cytotoxic chemotherapies or even targeted molecular inhibitors,^3^ while the latter is likely to reflect the delayed effect of the cancer vaccines mentioned earlier, which necessitates larger patient populations to achieve adequate statistical power.

## Discussion

In this study, we have analyzed the characteristics of all the cancer vaccine trials registered in the US Clinical Trials Database before 2013, corresponding to 955 trials. The chosen database is the most comprehensive trial database available.^32^ Such a methodology has been previously applied by ourselves and others to characterize the trial landscape in traumatic brain injury,^30^ as well as nephrology^33^ and oncology in general.^34^

## Overall trial characteristics

A longitudinal analysis of cancer vaccine trials revealed a peak in 2008 followed by a decline (Figure 2A). While this could be due to the global reasons across the cancer research sector, or perhaps the entire clinical trial landscape, this is made unlikely by the fact that a similar peak in trial count could not be observed when nonvaccine interventional cancer trials were analyzed. Instead, this finding suggests that there is indeed a real and significant decline in the translational effort for cancer vaccines; this is probably a reflection of the poor therapeutic yield of the field as earlier discussed and suggests that a greater urgency is necessary in identifying and addressing the underlying causes.

An analysis of the trials by phase obtained a distribution that was not dissimilar to that obtained by others who have analyzed cancer interventional trials.^35^ These data are in line with previous data that had shown the response rate in early phase cancer vaccine trials to be similar to the lower end of the response rates observed in early phase interventional cancer trials.^4^ This suggests that, in their current design, Phase II cancer vaccination trials excessively overestimate therapeutic efficacy. Consequently, although it has been argued that the selection of trial endpoints underestimates the benefit of the cancer vaccines,^4^,^27^ the precise effects of trial endpoints on response rates are likely to be more complex.

## Nature of vaccination approach

Our analyses of antigen formulation revealed that 25% of the trials included a wide range of tumor-associated antigens (Figure 3), and 32% of the trials included a delivery vector (Figure 4). This lends quantitative support to earlier suggestions that the contributing factors to the poor efficacy of the cancer vaccines include (i) vaccination against too few antigens, thus making tumor escape possible,^10^ as well as (ii) the failure to deliver antigens into the cytosol of antigen-presenting cells so as to enable major histocompatibility complex class I presentation and thus a cytotoxic T-lymphocyte response.^11^

Interestingly, the previously mentioned proportions have remained approximately constant over time. This suggests that – despite calls to modify the antigen formulation – the approach to clinical trials remains conservative on these questions. This may be due to significant logistical difficulties in the production of such vaccines, in spite of their theoretical advantages, most notably for autologous vaccine formulations that require the preparation of patient-specific vaccines from their tumor material and/or peripheral blood APCs.^11^

We have observed that a wide spectrum of adjuvants was used, indicating that adjuvants account for a sizable proportion of variation between vaccine formulations (Table 1). This observation implies that the optimal adjuvant is still uncertain, and it also supports earlier observations of a nontargeted approach across the field toward adjuvant selection, which may be hindering the identification of the optimal adjuvant.^36^ Indeed, the necessity of carefully testing the effect of adjuvants was highlighted by a recent trial, which showed that the addition of granulocyte macrophage colony-stimulating factor to an melanoma tumor cell vaccine reduced T-cell responses and OS at 2 years.^36^ Thus, the lack of a systematic approach to adjuvant testing may contribute to the poor efficacy of the cancer vaccines in general.

## Existing trial methodology

The most frequently studied cancers are melanoma and cancers of the cervix, breast, lung, and prostate (Figure 5A). These data are broadly in line with that of Dayoub and Davis, who performed a cross-sectional analysis of the therapeutic tumor vaccine trials registered from January–May 2011.^37^

The frequency with which breast, lung, and prostate cancers are studied is likely to reflect their high incidence and contribution to annual mortality,^37^ while the study of melanoma and cervical cancers is likely to be driven – at least in part – by their perceived tractability to vaccination strategies, due to the immunogenicity of melanomas and the infectious etiology of cervical cancers. While renal cancers have been considered immunogenic as well, the low frequency with which they have been studied may be due to disappointing results from a number of adjuvant clinical trials of autologous renal cell carcinoma vaccines in the late 1990s to the early 2000s.^8^

This analysis of the cancer types studied provides some support for the argument that the translational failures of cancer vaccines are due to efforts being directed at the common cancers less amenable to vaccine therapy. Further support can be obtained from the observation that – apart from sipuleucel-T – the other cancer vaccines receiving approval are targeted at cervical cancer, malignant melanoma, or renal carcinoma.

Interestingly, however, the proportion of trials involving melanoma patients is on the decrease. This may reflect a more nuanced and less empirical understanding of cancer immunogenicity, for instance through the identification of more TAAs^38^ across different cancer types. An additional explanation for this relative decline of melanoma vaccine trials could be the recent success of immune checkpoint inhibition, eg, ipilimumab,^6^ in treating metastatic melanoma. Both these developments may have resulted in translational efforts being refocused away from melanoma, which may subsequently lead to a further dilution and decline of therapeutic success in the field.

Also, this study has revealed that a relatively small proportion of the translational effort has been directed at preventive cancer vaccines. Most of these vaccines are directed against HPV (Figure 6B). Although the proportion of cancer vaccine trials conducted in the preventive setting has been increasing, it is doubtful whether this overall trend will continue, given the presence of a noticeable peak in trials initiated in 2007, and the fact that two vaccines against HPV strains, Cervarix^®^ (GlaxoSmith-Kline plc, London, UK) and Gardasil^®^ (Merck & Co, Inc., Whitehouse Station, NJ, USA), have already received regulatory approval.

Further efforts in the field may be driven by the development of vaccines against other infectious agents, for instance, *Helicobacter pylori* (NCT00613665) in the context of gastric carcinoma or gastric mucosa-associated lymphoid tissue lymphoma. However, the economic drivers of these translational efforts may be weaker, given the fact that some, if not many, of the infectious causes of cancers have a higher incidence in poorer countries, eg, *H. pylori* and *Schistosoma haematobium*.^21^

For the remaining trials that were conducted with the aim of treatment rather than prevention, we have shown that 67% of the trials assessed vaccine efficacy exclusively in the adjuvant setting, with a further 26% including at least some patients free of macroscopic disease (Figure 6C). This suggests that the lack of therapeutic efficacy of candidate vaccines cannot be attributed to the immunosuppressive effects of the tumor in situ. The local immunosuppressive effect of the stroma in micrometastases, however, is potentially causative and – in the preclinical setting – is currently actively researched. Also, these data cannot exclude the possibility that vaccine efficacy is attenuated by immunosuppressive effects of the tumor that persist even after resection, as has been demonstrated in a mice melanoma model.^39^ Indeed, poor vaccine responses in the adjuvant setting may even perhaps be attributable to the long-term immunosuppressive effects of adjuvant cytotoxic chemotherapy,^2^,^40^ a valuable question that can be addressed in future clinical trial analyses.

With regard to trial endpoints, our data suggest some further explanations regarding the use of existing endpoints that may contribute to the high rate of late phase failures. First, a minority of Phase III trials do not assess OS, but depend instead on endpoints, such as PFS and disease-free survival, even though several immunotherapies, (eg, sipuleucel-T and ipilimumab), have demonstrated increases in OS without attendant increases in the PFS or related measures.^3^ Second, in 42% of Phase II trials, no survival-related endpoint was measured, suggesting that a significant proportion of candidate vaccines enter late phase trials on the basis of radiological evidence of tumor response, a surrogate measure that is known to be problematic for immunotherapies.^41^

Most importantly from a translational research point of view, this study has also revealed that only a small proportion of late phase trials assess the immune response to the tumor vaccine under investigation (Figure 7A). We suggest that Phase II and especially Phase III trials should include objective immune response analyses more frequently to facilitate translational science and a better understanding of positive and negative trial outcomes, especially in the context of a high proportion of failed late stage clinical trials. Moreover, immunological data from the late phase trials will be vital to enabling the optimal use of cancer vaccines in clinical practice postlicensing, as given the aforementioned problems with assessing vaccine response radiologically, some surrogate of the clinical benefit is required to prognosticate and plan for additional therapies, which may be therapeutic or palliative.^3^

Trial design can be adjusted to take into account the logistical demands of immunological endpoint assessment, including assessing these endpoints in a subgroup of patients to reduce costs or only in selected participating tertiary referral centers, where the necessary technical expertise is available. Regardless, beginning to assess immunological endpoints now will enable the development of the necessary clinical trial infrastructure to do so more reliably and cost effectively in the future.

## Conclusion

By characterizing the landscape of interventional clinical vaccine trials, this study has revealed declining numbers of trials initiated since 2008; there is a need for greater urgency in removing the obstacles to the clinical translation of experimental vaccines.

Our data have demonstrated that only in a minority of trials are vaccines that incorporate a wide range of tumor antigens or utilize vectors for antigen delivery assessed, providing quantitative support for the hypotheses that these characteristics of experimental vaccines are impeding clinical translation. We have also observed a significant lack of consistency in terms of the adjuvants employed in the various trials, including Phase III trials – suggesting that cancer vaccines, in general, still lack effective adjuvants. In terms of the clinical trial methodology, our data reveal that while melanoma is the most common cancer studied, significant clinical efforts are being directed at common cancers not regarded as particularly immunogenic. Also, we have confirmed the observation that only a minority of cancer vaccines is used prophylactically, predominantly HPV vaccines. We have demonstrated that, in addition to this, the majority of therapeutic cancer vaccines are trialed in the adjuvant setting, suggesting that disease volume has little impact on vaccine efficacy. Longitudinally, the overall picture is generally one of stasis, with minimal evolution of the trial landscape in spite of various calls to the contrary. Finally, considering both the finding of a surprisingly high proportion of Phase III trials and the observation that 42% of Phase II trials did not utilize any survival-related endpoints, we suggest that a failure to adequately estimate therapeutic efficacy in Phase II trials is contributing, at least in part, to the high rate of translational failure in the late phase trials. We also note the relative paucity of mechanistic immunological endpoints in the Phase III trials, which – if not rectified – is likely to hinder translational efforts in the field as well as the optimal clinical use of approved vaccines.

## Supplementary material

**Table S1:** Details of Phase III trials commencing between 2008 and 2012

NCT #	Formulation	Title	First received	Start year	Primary completion	Enrollment	Status
NCT01072981	Algenpantucel-L	Immunotherapy Study for Surgically Resected Pancreatic Cancer	2010	2010	2014	722	Recruiting
NCT00676507	Belagenpumatucel-L	Phase III Lucanix™ Vaccine Therapy in Advanced Non-small Cell Lung Cancer (NSCLC) Following Front-line Chemotherapy	2008	2008	2012	506	Active, not recruiting
NCT01479244	E75 peptide plus GM-CSF vaccine	Efficacy and Safety Study of NeuVax™ (Nelipepimut-S or E75) Vaccine to Prevent Breast Cancer Recurrence	2011	2011	2015	700	Recruiting
NCT01015443	Emepepimut-S	Cancer Vaccine Study for stage III, Unresectable, Non-small Cell Lung Cancer (NSCLC) in the Asian Population	2009	2009	2016	420	Recruiting
NCT00925548	Emepepimut-S	A Study of Stimuvax ^®^ in Combination With Hormonal Treatment Versus Hormonal Treatment Alone for First-line Therapy of Endocrine-sensitive Advanced Breast Cancer	2009	2009	2010	42	Terminated
NCT01322490	Fowlpox-PSA-TRICOM vaccine, Vaccinia-PSA-TRICOM vaccine	A Randomized, Double-blind, Phase 3 Efficacy Trial of PROSTVAC-V/F +/− GM-CSF in Men With Asymptomatic or Minimally Symptomatic Metastatic Castrate-Resistant Prostate Cancer	2011	2011	2015	1,200	Recruiting
NCT01579188	GV1001	Study of the Telomerase Vaccine GV1001 to Treat Patients With Inoperable Stage III Non-small Cell Lung Cancer	2012	2012	2016	600	Not yet recruiting
NCT01047345	HPV – V503	A Study of V503 Vaccine in Females 12–26 Years of Age Who Have Previously Received GARDASIL™ (V503-006 AM1)	2010	2010	2011	924	Completed
NCT00943722	HPV – V503	A Study of V503 in Preadolescents and Adolescents (V503-002 EXT1 EXT2)	2009	2009	2011	3,074	Active, not recruiting
NCT01254643	HPV – V503	A Study of the Safety, Tolerability, and Immunogenicity of V503 Administered to 9- to 15-Year-Old Japanese Girls (V503-008)	2010	2011	2013	100	Active, not recruiting
NCT01073293	HPV – V503	A Study of V503 Vaccine Given Concomitantly With REPEVAX™ in 11 to 15 Year Olds (V503-007 AM1)	2010	2010	2011	1,054	Completed
NCT00988884	HPV – V503	A Study of V503 Given Concomitantly With Menactra™ and Adacel™ in 11 to 15 Year Olds (V503-005) (COMPLETED)	2009	2009	2011	1,245	Completed
NCT01735006	HPV 16/18 L1 viruslike particle vaccine	Efficacy and Immunogenicity Study of Recombinant Human Papillomavirus Bivalent Type 16/18 Vaccine	2012	2012	2015	6,000	Recruiting
NCT00637195	HPV 16/18 L1 viruslike particle/AS04 vaccine	Immunogenicity and Safety of a Commercially Available Vaccine Co-administered With GSK HPV Vaccine (580299)	2008	2008	2009	152	Completed
NCT01381575	HPV 16/18 L1 viruslike particle/AS04 vaccine	Evaluation of Immunogenicity and Safety of Two 2-dose Human Papillomavirus (HPV) Vaccine Schedules in 9–14 Year Old Girls	2011	2011	2014	1,428	Active, not recruiting
NCT00799825	HPV 16/18 L1 viruslike particle/AS04 vaccine	Safety Study of GSK Biologicals’ Human Papillomavirus Vaccine in 580299/008 Subjects From Canada or the US	2008	2009	2012	1,000	Completed
NCT00849381	HPV 16/18 L1 viruslike particle/AS04 vaccine	Safety Study of GSK Biologicals’ Human Paillomavirus Vaccine in 580299/008 Subjects from Brazil, Taiwan or Thailand	2009	2009	2012	1,239	Completed
NCT00779766	HPV 16/18 L1 viruslike particle/AS04 vaccine	Efficacy, Immunogenicity and Safety of GSK Biologicals’ HPV GSK 580299 Vaccine in Healthy Chinese Female Subjects	2008	2008	2011	6,051	Active, not recruiting
NCT01190189	HPV 16/18 L1 viruslike particle/AS04 vaccine	Safety Evaluation of the GSK-580299 Vaccine in Women From the Control Group in the Primary NCT00294047 Study	2010	2011	2015	600	Recruiting
NCT00929526	HPV 16/18 L1 viruslike particle/AS04 vaccine	Extension Study of the Efficacy of the GSK 580299 Vaccine in Japanese Women Vaccinated in the Primary NCT00316693 Study	2009	2009	2011	752	Completed
NCT01249365	HPV 16/18 L1 viruslike particle/AS04 vaccine	The Safety Evaluation of the GSK-580299 Vaccine in Women From the Control Group in the Primary NCT00294047 Study	2010	2011	2015	465	Recruiting
NCT01627561	HPV 16/18 L1 viruslike particle/AS04 vaccine	Safety and Immunogenicity of GlaxoSmithKline (GSK) Biologicals’ Human Papillomavirus Vaccine in Healthy Female Children	2012	2012	2016	1,000	Recruiting
NCT01277042	HPV 16/18 L1 viruslike particle/AS04 vaccine	Study to Assess Immune Responses and Safety of the GSK-580299 Vaccine in Healthy Women (26 to 45 Years)	2011	2011	2012	1,212	Completed
NCT00811798	HPV 16/18 L1 viruslike particle/AS04 vaccine	Safety Study of GSK Biologicals’ HPV Vaccine (GSK-580299) in Healthy Female Subjects	2008	2009	2010	92	Completed
NCT00652938	HPV 16/18 L1 viruslike particle/AS04 vaccine	Evaluation of Immunogenicity and Safety of Human Papillomavirus (HPV) Vaccine Co-administered With Another Vaccine in Healthy Female Subjects	2008	2008	2009	744	Completed
NCT01190176	HPV 16/18 L1 viruslike particle/AS04 vaccine	Gynaecological Follow-up of a Subset of HPV-015 ( NCT00294047) Study Subjects	2010	2011	2018	1,500	Recruiting
NCT00877877	HPV 16/18 L1 viruslike particle/AS04 vaccine	Evaluation of Long-term Immunogenicity and Safety of a Human Papillomavirus (HPV) Vaccine in Healthy Female Subjects	2009	2009	2010	529	Active, not recruiting
NCT00947115	HPV 16/18 L1 viruslike particle/AS04 vaccine	Evaluation of Long-term Immunogenicity and Safety of a Human Papillomavirus (HPV) Vaccine in Healthy Female Subjects	2009	2009	2010	666	Active, not recruiting
NCT01418937	HPV 16/18 L1 viruslike particle/AS04 vaccine	Safety Evaluation of a Human Papillomavirus (HPV) Vaccine in Healthy Female Control Subjects From the GSK HPV 023 Study	2011	2012	2014	220	Recruiting
NCT00937950	HPV 16/18 L1 viruslike particle/AS04 vaccine	Gynaecological Follow-up of a Subset of 580299/008 ( NCT 00122681) Study Subjects	2009	2009	2014	2,500	Recruiting
NCT01651949	HPV vaccine V503	Multivalent HPV (Human Papillomavirus) Vaccine Study in 16- to 26-Year Old Men and Women (V503-003 AM5)	2012	2012	2014	2,500	Recruiting
NCT01265901	IMA901	IMA901 in Patients Receiving Sunitinib for Advanced/Metastatic Renal Cell Carcinoma	2010	2010	2014	330	Active, not recruiting
NCT01546571	POL-103A	Study of a Melanoma Vaccine in Stage llb, llc, and III Melanoma Patients	2012	2012	2016	1,059	Recruiting
NCT00693342	Polyvalent antigen-KLH conjugate vaccine	Vaccine Therapy and OPT-821 or OPT-821 Alone in Treating Patients With Ovarian Epithelial Cancer, Fallopian Tube Cancer, or Primary Peritoneal Cancer in Complete Remission	2008	2008	2012	0	Withdrawn
NCT01245764	Quadrivalent HPV (types 6, 11, 16, 18) recombinant vaccine	GARDASIL™ Study in Healthy Females Between 9 and 26 Years of Age in Sub-Saharan Africa (V501-046)	2010	2011	2013	250	Completed
NCT01461096	Quadrivalent HPV (types 6, 11, 16, 18) recombinant vaccine	Evaluating the Effectiveness of the Quadrivalent Human Papillomavirus (HPV) Vaccine at Preventing Anal HPV Infection in HIV-Infected Men and Women	2011	2012	2015	564	Recruiting
NCT01375868	Quadrivalent HPV (types 6, 11, 16, 18) recombinant vaccine	Effect of Vaccination in Patients With Recurrent Respiratory Papillomatosis	2011	2011	2017	50	Recruiting
NCT00496626	Quadrivalent HPV (types 6, 11, 16, 18) recombinant vaccine	An Immunogenicity and Safety Study of Gardasil ^®^ in Chinese Subjects (V501-030) (COMPLETED)	2007	2008	2009	600	Completed
NCT00964210	Quadrivalent HPV (types 6, 11, 16, 18) recombinant vaccine	Protecting Young Special Risk Females From Cervical Cancer Through Human Papilloma Virus (HPV) Vaccination	2009	2008	2010	240	Completed
NCT01460472	Racotumomab	Immunotherapy With Racotumomab in Advanced Lung Cancer	2011	2010	2015	1,082	Recruiting

**Abbreviations:** HPV, human papillomavirus; NCT, National Clinical Trial; GM-CSF, granulocyte macrophage colony-stimulating factor.

## Figures and Tables

**Table 1:** Vaccines investigated in Phase III trials commencing between 2008 and 2012, (n=40)

Vaccine	Description	Adjuvants	Setting	NCT #
Algenpantucel-L	Alpha-1,3-galactosyltransferase-expressing allogeneic pancreatic tumor cell vaccine	None	Adjuvant	NCT01072981
Belagenpumatucel-L	Contains NSCLC tumor cells	TGF-β2 antisense	Adjuvant	NCT00676507
E75 peptide + GM-CSF vaccine	HLA A2/A3-restricted HER-2/neu peptide vaccine	GM-CSF	Prevention	NCT01479244
Emepepimut-S	Liposomal BLP25 vaccine	MPL	Adjuvant	NCT01015443
Fowlpox-PSA-TRICOM vaccine	Recombinant fowlpox vaccine encoding prostate-specific antigen	TRICOM (B7.1, ICAM-1 and LFA-3)	Adjuvant	NCT01322490
GV1001	Contains telomerase peptide	GM-CSF	Adjuvant	NCT00925548
HPV 16/18 L1 viruslike particle vaccin0065	Different formulation from Cervarix developed by Xiamen Innovax Biotech	Alum	Prevention	NCT01735006
HPV 16/18 L1 viruslike particle/AS04 vaccine		AS04 (Alum +MPL)	Prevention	NCT00637195 NCT01381575 NCT00799825 NCT00849381 NCT00779766 NCT01190189 NCT00929526 NCT01249365 NCT01627561 NCT01277042 NCT00811798 NCT00652938 NCT01190176 NCT00877877 NCT00947115 NCT01418937 NCT00937950
HPV vaccine V503	Contains L1 capsid proteins for nine HPV strains	Amorphous aluminum hydroxyphosphate sulfate	Prevention	NCT01651949 NCT01047345 NCT00943722 NCT01254643 NCT01073293 NCT00988884
IMA901	Contains ten renal cell carcinoma associated peptide antigens	GM-CSF	Nonadjuvant	NCT01265901
POL-103A	Contains purified antigens from melanoma cell lines	Alum	Adjuvant	NCT01546571
Polyvalent antigen-KLH conjugate vaccine	Contains globo H, GM2 ganglioside, Tn-MUC1, TF, and sTn	OPT-821 (purified, natural saponin)	Adjuvant	NCT00693342
Quadrivalent HPV (types 6, 11, 16, 18) recombinant vaccine		Amorphous aluminum hydroxyphosphate sulfate	Prevention (4), precancerous (1)	NCT00964210 NCT01245764 NCT01461096 NCT01375868 NCT00496626
Racotumomab	Anti-P3 antibody idiotype monoclonal antibody 1E10	Alum	Adjuvant	NCT01460472
Vaccinia-PSA-TRICOM vaccine	Recombinant vaccinia vaccine encoding prostate-specific antigen	TRICOM (B7.1, ICAM-1 and LFA-3)	Adjuvant	NCT01322490

**Abbreviations:** HPV, human papillomavirus; GM-CSF, granulocyte macrophage colony-stimulating factor; MPL, monophosphoryl lipid A; TGF-β, transforming growth factor beta; NCT, National Clinical Trial; NSCLC, non-small-cell lung cancer.
